# The Lesioned Spinal Cord Is a “New” Spinal Cord: Evidence from Functional Changes after Spinal Injury in Lamprey

**DOI:** 10.3389/fncir.2017.00084

**Published:** 2017-11-06

**Authors:** David Parker

**Affiliations:** Department of Physiology, Neuroscience and Development, University of Cambridge, Cambridge, United Kingdom

**Keywords:** spinal cord injury, lamprey, neuromodulation, regeneration, plasticity

## Abstract

Finding a treatment for spinal cord injury (SCI) focuses on reconnecting the spinal cord by promoting regeneration across the lesion site. However, while regeneration is necessary for recovery, on its own it may not be sufficient. This presumably reflects the requirement for regenerated inputs to interact appropriately with the spinal cord, making sub-lesion network properties an additional influence on recovery. This review summarizes work we have done in the lamprey, a model system for SCI research. We have compared locomotor behavior (swimming) and the properties of descending inputs, locomotor networks, and sensory inputs in unlesioned animals and animals that have received complete spinal cord lesions. In the majority (∼90%) of animals swimming parameters after lesioning recovered to match those in unlesioned animals. Synaptic inputs from individual regenerated axons also matched the properties in unlesioned animals, although this was associated with changes in release parameters. This suggests against any compensation at these synapses for the reduced descending drive that will occur given that regeneration is always incomplete. Compensation instead seems to occur through diverse changes in cellular and synaptic properties in locomotor networks and proprioceptive systems below, but also above, the lesion site. Recovery of locomotor performance is thus not simply the reconnection of the two sides of the spinal cord, but reflects a distributed and varied range of spinal cord changes. While locomotor network changes are insufficient on their own for recovery, they may facilitate locomotor outputs by compensating for the reduction in descending drive. Potentiated sensory feedback may in turn be a necessary adaptation that monitors and adjusts the output from the “new” locomotor network. Rather than a single aspect, changes in different components of the motor system and their interactions may be needed after SCI. If these are general features, and where comparisons with mammalian systems can be made effects seem to be conserved, improving functional recovery in higher vertebrates will require interventions that generate the optimal spinal cord conditions conducive to recovery. The analyses needed to identify these conditions are difficult in the mammalian spinal cord, but lower vertebrate systems should help to identify the principles of the optimal spinal cord response to injury.

## Introduction

Recovery from a spinal cord injury (SCI) in mammals, including humans, is minimal. Despite hopes, and claims, for effective therapies, the outlook for targeted improvement remains poor. Injury is associated with the loss of motor, autonomic, and sensory function, as well as the development of deleterious effects like spasticity and neuropathic pain (Störmer et al., 1997; Kuner, 2010). While several approaches that target different aspects of the spinal cord are being used to try to overcome the effects of injury, there is currently no effective treatment. Fong et al. (2009) write, “consumed with individual pieces of the puzzle, we have failed as a community to grasp the magnitude of the sum of our findings.”

Treatments for SCI have been categorized as those that rescue, reactivate, and rewire (Ramer et al., 2014). Rescue involves early interventions that try to reduce secondary damage after the initial injury. Reactivation attempts to activate spared systems after a SCI using rehabilitative, pharmacological, or electrical stimulation. Reactivation may not be the correct term, as it suggests a need to restore activity, but once the period of spinal shock has passed activity can be excessive and lead to the development of deleterious effects (e.g., Kuner, 2010; D’Amico et al., 2014). Rather than reactivation, recalibration may be needed to restore the correct balance of activity. Rewiring is the dominant approach in SCI research, and attempts to regenerate the connections damaged by the lesion (see Ramer et al., 2014). This is an obvious strategy given that SCI effects are caused by the loss of these inputs. Hundreds of preclinical studies have reported functional improvements after SCI using regenerative approaches (Ramer et al., 2014). However, while regeneration or regrowth can lead to significant functional improvements, there is often a poor correlation between regeneration and recovery (see for example, Blackmore et al., 2012; Granger et al., 2012; Lu et al., 2012; Zukor et al., 2013), and the replication of any effect is also often limited (Steward et al., 2012). Tuszynski and Steward (2012) wrote, “every report of a treatment that produced dramatic regeneration and recovery of function after SCI has failed to stand the test of time and scrutiny.” Recently, a clinical trial using stem cells, the latest approach claimed to offer a potential treatment for SCI, was terminated due to the lack of any improvement (Servick, 2017). In contrast to complete SCI, considerable functional recovery can occur with incomplete lesions (although this paradoxically leads to more severe neuropathic pain; Störmer et al., 1997). Potassium channel blockers have been used to improve conduction in spared axons across lesion sites, but clinical trials again failed to show significant motor, sensory, or autonomic improvements (Cardenas et al., 2006). Lack of replication is of course not limited to SCI research (Hartung, 2013).

Issues of replication in studies that attempt to promote regeneration or alter the effectiveness of spared axons may reflect the fact that the descending input to the spinal cord is only one of several factors needed to generate effective outputs. The most obvious is that while promoting regeneration or manipulating the properties of spared axons seems necessary and reasonable, these approaches can only be beneficial if they lead to functionally appropriate interactions below the lesion site.

Locomotor networks below lesion sites remain after injury, and can control complex movements if they receive appropriate inputs. This has been examined with epidural stimulation of the spinal cord in experimental and clinical studies (“electro-enabling motor control,” Edgerton and Harkema, 2011). Edgerton and Harkema (2011) say that the results of epidural stimulation call for a change in SCI recovery strategies from the focus on regeneration and repair to instead addressing how best to activate remaining circuitry. Stimulation is suggested to work by increasing spinal locomotor network excitability. However, this is probably too simplistic, as network excitability cannot go unchecked but needs to be balanced by appropriate levels of presynaptic and postsynaptic inhibition (“balanced inhibition”; Berg et al., 2007). The balanced inhibition model suggests that rather than the assumed alternating phases of reciprocal excitation and inhibition during locomotion, network excitation occurs on a background of co-varying inhibition, resulting in a critical state that allows excitatory phases to be rapidly modulated to generate a reliable but flexible motor output. If this is a general mechanism in spinal cord networks (Berg et al., 2007), as it is in other networks (Populin, 2005; Magnusson et al., 2008; Atallah and Scanziani, 2009; Yizhar et al., 2011; Atallah et al., 2012; Xue et al., 2014), then simply adding excitation could perturb this balance and cause functional impairments (e.g., dystonia; Quartarone et al., 2008), or excitotoxic damage and cell death. Any system of feedforward and feedback pathways requires the appropriate interactions between components for its effective activation, and this will also be necessary for any intervention after SCI. Understanding the nature of spinal cord excitation and inhibition rather than assuming a reciprocal pattern is thus an outstanding basic question of general importance, that should allow for more effective spinal cord stimulation regimes.

This review summarizes published and unpublished work we have done on SCI in the lamprey, a lower vertebrate model system. The emphasis was initially on changes in the sub-lesion locomotor network, but has been extended to include supra-lesion changes, sensory feedback, and the functional properties of regenerated synapses. The data suggests that even though locomotor behavior returns to pre-lesion levels, this reflects changes in spinal cord function and plasticity at all levels. Several individual effects have been identified that correlate with the degree of recovery. However, the data suggests that neither regenerated inputs, locomotor networks, nor sensory inputs are both necessary and sufficient for recovery, and that rather than a single aspect, recovery requires appropriate changes and interactions within and between different components of the spinal cord motor system.

## The Lamprey As A Model for Spinal Cord in Injury

The lamprey is a lower vertebrate model system for analyses of spinal cord function, and regeneration and recovery after SCI (Rovainen, 1979; Cohen et al., 1988; McClellan, 1994; Grillner et al., 1998; Buchanan, 2001). Axonal regeneration across a lesion site and functional recovery in lamprey occur by 8 weeks after a complete spinal cord transection (Selzer, 1978; Rovainen, 1979; Wood and Cohen, 1979). Regeneration has been studied extensively, and seems a necessary condition for recovery (McClellan, 1994; Cohen et al., 1988; Smith et al., 2011; Rasmussen and Sagasti, 2017).

Lower vertebrates could claim to offer support for the focus on regeneration after SCI: lower vertebrates regenerate and recover locomotor function, higher vertebrates don’t regenerate and don’t recover locomotor function; thus, regeneration equals recovery. However, this is a logical fallacy that confuses necessity with sufficiency: regeneration can be necessary for recovery without being sufficient. Several aspects complicate the link between regeneration and recovery in lamprey, but of importance for the discussion here is that regeneration is never complete; regenerated axons grow short distances and project to ectopic locations; and regenerated synapses are sparser and smaller (see Selzer, 1978; Rovainen, 1979; Wood and Cohen, 1981; Yin and Selzer, 1983; Davis and McClellan, 1994; Armstrong et al., 2003; Shifman et al., 2008; Oliphint et al., 2010; Jin et al., 2016). Similar effects are seen with regeneration in other lower vertebrates (Bernstein and Gelderd, 1973). Regeneration is thus not repair in the sense that the spinal cord is restored to the pre-lesion state. To claim that regeneration equals recovery requires the assumption that at least 30% of the descending input in the unlesioned spinal cord is degenerate or redundant (the maximum extent of regeneration in lamprey is ∼70%; McClellan, 1994), and that the precise location of inputs is unimportant. Our hypothesis was that regeneration was necessary, but not sufficient, for recovery, and that compensatory changes in the spinal cord also influence behavioral recovery.

The motor system can crudely be split into three components: descending inputs from the brain, sensory inputs, and locomotor networks. While these are often studied separately, motor outputs will ultimately reflect their interactions through feedforward and feedback pathways (Pearson, 2000). Lesioning disrupts these interactions, and recovery thus needs to overcome this disruption. These interactions could generate a continuum of effects, from regeneration alone being sufficient, to a stage where recovery depends solely on other factors. The relative balance between components, where each modifies its activity to match that of the others, would be an example of compensatory plasticity. We have started to examine how each of these components is altered after injury in lamprey.

The lesion is a critical variable in studies of regeneration. SCI models with clinical relevance are better represented by contusion injuries (Cheriyan et al., 2014). However, these cause variable partial lesions that complicate the characterization of changes after injury (e.g., regeneration; Geoffroy and Zheng, 2014). While a complete transection of the spinal cord is less clinically relevant than a contusion injury, as clinical relevance is not a factor in lower vertebrate analyses we have used complete transection to that remove the variability introduced by partial lesions.

Finally, functional recovery after SCI is greater in younger patients than adults. This is also seen in various animal systems despite the marked developmental differences in spinal cord maturity and function across species. This presumably reflects the greater plasticity of the immature spinal cord (Pape, 2012). We have used two developmental stages, larval and juvenile adult lampreys, to examine how systems in different developmental (and functional) states adapt to injury.

## Compensatory Plasticity

A major challenge to any intervention after SCI is that nervous system components are altered, even when not directly affected by the injury (see Dunlop, 2008). These changes can be effectively instant (e.g., diaschisis; Carrera and Tononi, 2014). Rather than being exceptional, diaschisis is expected in any feedforward and feedback system, as even highly localized perturbations will necessarily affect other components. In addition, slower secondary changes (minutes to weeks) can develop after injury (e.g., inflammation and demyelination). Finally, compensatory plasticity can develop that responds to the initial injury, diaschisis, or the secondary changes (Turrigiano, 1999; Davis and Bezprozvanny, 2001; Frank, 2014). A SCI will thus not just disconnect the spinal cord, but will result in a period of considerable flux, the spinal cord ultimately settling into a new functional state.

Compensatory effects have been known for many years (e.g., denervation supersensitivity described by Walter Cannon in the 1930’s; see Thesleff and Sellin, 1980). These effects have been termed “homeostatic plasticity,” as they can develop in ways that will oppose a perturbation (see Marder and Goaillard, 2006). However, the changes are not necessarily intuitive. For example, inhibition increases when excitation is reduced in cortical (Maffei et al., 2006) and hippocampal slices (Echegoyen et al., 2007). This is the opposite to that expected of a homeostatic response, which should trigger changes that reduce inhibition or increase excitation. Increasing inhibition can lead to an increase in excitation through disinhibition. However, even if inhibition alone was increased this would not necessarily be “non-homeostatic,” as homeostasis is not simply synonymous with negative feedback clamping of parameter values (see Buchman, 2006). Homeostasis can also include feedforward prediction, variable and changing set-points, and hierarchical control that can trigger variable changes at different levels (including positive feedback and amplification of perturbations), to ultimately maintain higher level function despite marked changes in lower level properties (Cannon, 1932). What may seem non-homeostatic at one level may ultimately be homeostatic at the system level.

There is evidence for a role for compensatory changes in recovery from stroke (Dancause and Nudo, 2011). Similar effects would have obvious relevance to recovery from SCI. Functional changes after SCI are demonstrated clinically by the appearance of spasticity once the period of spinal shock has passed (Bennett, 2008). They have also been shown experimentally. For example, cats that had a spinal cord hemisection followed by a complete transection 64–80 days later learnt to step on a treadmill faster than those that received a single complete transection (Frigon et al., 2009). Courtine et al. (2008) showed a similar effect using two staggered hemisections on different sides of the mouse spinal cord: mice quickly recovered locomotion and weight support after the second lesion even though all descending inputs were disrupted. A spinal hemisection in rats initially reduced synaptic inputs to motor neurons, but these inputs subsequently recovered, albeit with a reduction of putative inhibitory synapses made onto the cell body (Nacimiento et al., 1995). There is also evidence for increased GABA and glycine levels after SCI (Tillakaratne et al., 2000), which could suggest an increase in inhibition (but see Boulenguez et al., 2010 for changes in inhibitory effects after SCI). These are just a few of very many examples, but while functional changes after SCI are endemic, the limited spontaneous recovery after SCI in mammals shows that on their own they are not sufficient for recovery. This may reflect the necessary requirement for communication between the brain and spinal cord, and the absence of an alternative pathway around a complete spinal lesion.

While compensatory or homeostatic plasticity could facilitate recovery, plasticity is not a ubiquitous phenomenon. Plasticity is claimed to be greater in “higher” centers than in the brainstem and spinal cord where functions have become relatively fixed during evolution (Delgado-Garcia, 2015; but see Wolpaw and Tennissen, 2001). However, the latter areas are claimed to have a greater potential for compensatory changes to maintain their more stereotyped functions (see Delgado-Garcia, 2015). If this is the case, it would support a focus on compensatory plasticity after SCI. Plasticity is also not necessarily a desirable phenomenon, but can also result in deleterious effects (neuropathic pain, autonomic dysreflexia; Störmer et al., 1997; Quartarone et al., 2008; Kuner, 2010). Avoiding these effects requires insight into the type of plasticity needed, and as inappropriately timed plasticity can be deleterious (e.g., Kozlowski et al., 1996; Scivoletto et al., 2005), when it should be evoked. This is especially important after SCI, where the wave of functional changes caused by the initial injury, diaschisis, secondary effects, and associated compensations, could result in state-dependent influences on plasticity mechanisms or interventions.

All of these functional and structural changes will alter processing within spinal cord networks. This has led to the claim that the spinal cord below a lesion site is a “new” spinal cord (Edgerton et al., 2001). This might account for the inconsistent effects of regeneration: even if restoration of the pre-lesion descending input was possible, it may or may not generate a pre-lesion output depending on the current functional state of the spinal cord. An optimal output after SCI requires understanding how spinal cord circuitry is changed by injury (which necessitates an understanding of the normal spinal cord), and how best to integrate any input with these changes. Given the difficulties of understanding mammalian spinal cord networks (Rybak et al., 2015), these analyses should be facilitated in lower vertebrate systems. While these are not clinically relevant (there are issues of clinical relevance with any animal model; Hartung, 2013), the insight obtained can provide general principles of spinal cord adaptations to injury.

## Behavioral Recovery in Lamprey

**FIGURE 1:**
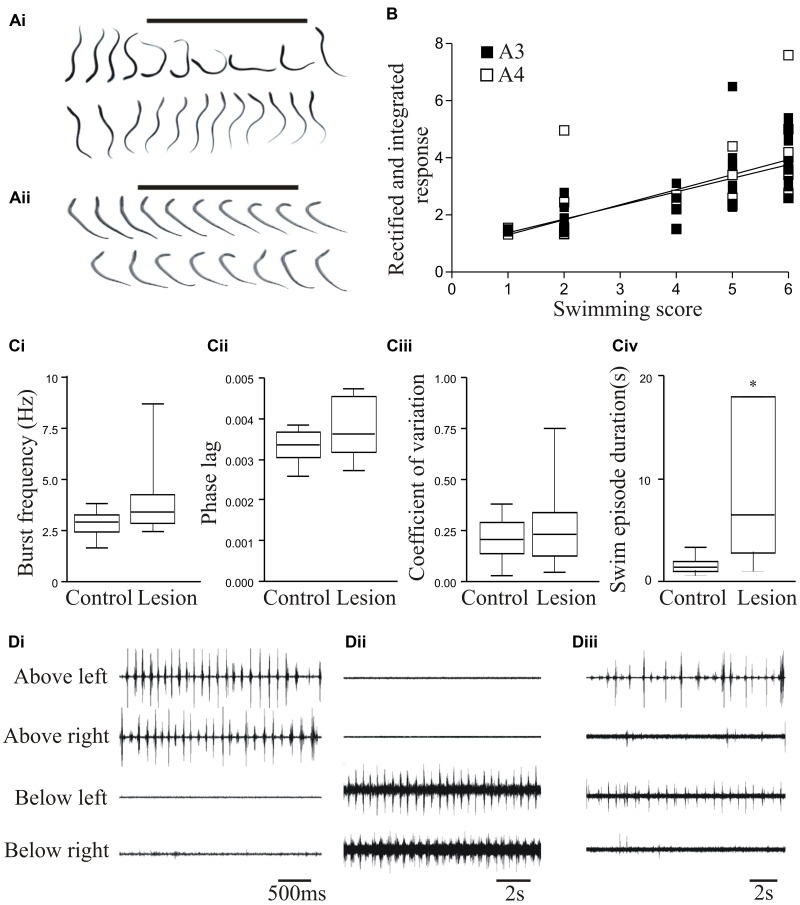
Behavioral recovery. **(Ai)** Video frames from an animal that recovered good locomotor function. The bar indicates a mechanical stimulus given to the body, and shows the coordinated movement away from the stimulus. **(Aii)** Swimming of an animal that failed to recover. **(B)** Graph showing the correlation of swimming performance with the degree of regeneration, assessed from ventral root responses below the lesion site ipsilateral (A3) and contralateral (A4) to stimulation above the lesion. **(Ci–Civ)** Graphs comparing the swimming performance in animals that recovered well with that of unlesioned animals: the only significant difference was the duration of a swimming episode. **(Di–Diii)** Example of myogram activity from animals that failed to recover locomotor function. Data from Hoffman and Parker (2011). Permission granted to reproduce. ^∗^Indicates statistical significance at *p* < 0.05.

To assess locomotor activity after recovery from lesioning we scored swimming behavior on a six-point scale based on Ayers (1989): stage 6 animals recover locomotion function (**Figure 1Ai**), while stage 1 animals fail to show any recovery (**Figure 1Aii**). In larval and juvenile adult lampreys that recovered well (stage 5/6; 80–90% of animals), swimming parameters (frequency, phase lag, and regularity) assessed from electromyograms did not differ significantly to unlesioned animals (**Figures 1Ai,Ci–Ciiii**; Hoffman and Parker, 2011; see also Cohen et al., 1988; McClellan, 1994), although the duration of a swimming episode was significantly increased (**Figure 1Civ**). Recovery thus restores normal locomotor function.

Stage 1/2 animals typically lacked regeneration, shown by the absence of activity in the spinal cord below the lesion site when stimulating above the lesion site (A3 and A4 responses; see **Figure 3Ai**). While stage 5/6 activity can occur without any regeneration (Cooke and Parker, 2009; Hoffman and Parker, 2011), there was a significant correlation between regeneration and recovery that supported a necessary role for regeneration (**Figure 1B**; Hoffman and Parker, 2011). However, there was variability and overlap in the extent of regeneration in stage 1/2 and stage 5/6 animals (see **Figure 1B**). This, together with the potential for recovery in some cases in the absence of regeneration, suggests the involvement of other factors in recovery.

While analyses typically focus on successful recovery, analyses of the small proportion of animals that fail to recover could be more informative. Poor recovery is associated with several behavioral defects (see Cohen et al., 1999; Hoffman and Parker, 2011), including the absence of activity above or below the lesion site (**Figures 1Di,Dii**); poor co-ordination across the lesion site associated with differences in the frequency or pattern of activity; and defects in the reciprocal coupling between the left and right sides of the spinal cord above and below the lesion site (**Figure 1Diii**). In an analysis of 16 stage 1/2 animals, none were able to generate activity below the lesion site, and 7 were also incapable of generating reliable alternating activity above the lesion site. Even when there was alternation above the lesion site, alternating activity was absent 20–80% of the time (Parker, unpublished observations). These defects need to be characterized as they could suggest influences on the success or failure of recovery. Their potential range and variability of defects means that this will require a large sample size.

## Fictive Locomotion

In addition to monitoring swimming in intact animals, spinal cord function can be examined in the isolated spinal cord using fictive locomotion evoked by glutamate-receptor agonists (e.g., McClellan, 1994). This allows locomotor networks to be examined in the absence of sensory and descending inputs. However, fictive activity can be difficult to evoke, and differs to normal locomotor activity (see Ayers et al., 1983; McClellan, 1990). This is not surprising given the nature of the preparation (no sensory or descending input, tonic application of glutamate receptor agonists; see Parker and Srivastava, 2013). In addition to Ayers et al. (1983), swimming was also compared in intact and *in vitro* preparations by Wallen and Williams (1984). Their analysis has been used to claim that “locomotor coordination can be generated by the isolated spinal cord in the same way as the intact behaving lamprey” (Grillner, 2003). However, Wallen and Williams state “that there was an experimental bias …. the sequences selected for analysis in each preparation were those that appeared least variable,” and they thus don’t contradict the conclusion of Ayers et al. (1983) and McClellan (1990) of disparities between fictive and actual locomotor activity.

While fictive locomotion offers experimental advantages, there are issues surrounding its use in SCI given the hypothesis that spinal cord perturbation evokes compensatory changes. Fictive activity varies between experiments for a given drug concentration, and shows a prolonged and variable development that requires preparations to be left for some hours before activity is examined (see Parker et al., 1998). Hemisection of the spinal cord can trigger significant changes in cellular and synaptic properties within ∼30 min (Hoffman and Parker, 2010). As the spinal cord is removed and tonically activated non-physiologically for some hours before experiments start, similar changes could also be introduced under fictive conditions. Fictive locomotion also assumes regular activity as the norm (this presumably influenced the data selection by Wallen and Williams, 1984). However, actual locomotion varies, the loss of variability being associated with motor pathologies (see Hausdorff, 2009). The highly regular activity sought and presented in fictive locomotion studies may thus reflect a pathological loss of complexity (Pincus, 1994; Bienenstock and Lehmann, 1998; Berg et al., 2007). Consistent with this, fictive activity differs to intact activity in being less susceptible to modification by sensory or descending inputs in both lamprey and mammals (see Fagerstedt and Ullén, 2001; Musienko et al., 2012; Hsu et al., 2016). These effects could reflect the tonic application of glutamate receptor agonists that contrasts the normal spatial and temporal variability of glutamate release, or the removal of descending and sensory inputs [see Cohen et al., 1996; Wang and Jung, 2002; in the tadpole spinal cord brainstem input is needed for normal locomotor activity (Li et al., 2009)]. As a result of these features, we have only examined locomotion in intact animals.

## Properties of Regenerated Axons

The morphology of regenerated axons have been studied extensively in lamprey. Analyses typically focus on the larger Muller reticulospinal axons, as these can be uniquely identified (Wood and Cohen, 1981; Hall et al., 1989; McHale et al., 1995; Oliphint et al., 2010; Zhang et al., 2011). These axons make functional connections below the lesion site, although electrical synapses are reduced (Wood and Cohen, 1981). Changes in the functional properties of these axons have been studied, with short-term changes in excitability developing that recover to unlesioned levels once regeneration has occurred (McClellan et al., 2008). However, while they are convenient models systems, the Muller axons may not be the most important in terms of recovery, as lesions of the medial column (where Muller axons project) do not abolish locomotion in functionally recovered animals (McClellan, 1990).

In contrast to the detailed anatomical analyses of regenerated axons (e.g., McClellan, 1994), the functional properties of regenerated synapses have received little attention (Oliphint et al., 2010). These properties will ultimately determine the functional effects of regenerated axons, and differences in these properties may explain some of the variability between regeneration and recovery (see above). We have examined the properties of regenerated synapses by making paired recordings from axons above the lesion site and motor neurons either above or below the lesion site (Cooke and Parker, 2009; Parker, unpublished data). Axons have been targeted in the lateral columns as re-lesion studies suggest that these regenerated inputs are important for functional recovery (McClellan, 1990), possibly due to their greater regenerative capacity (Chen et al., 2017). These axons are smaller than the medial column Muller axons, and stable long-term recordings are not as routine. Connections in unlesioned spinal cords (*n* = 42) and above (*n* = 22) and below a lesion site (*n* = 14) were examined in response to presynaptic stimulation at 20 Hz to characterize the initial and activity-dependent properties of the connections (see Parker, 2000). The data presented is from ongoing analyses. Because most animals recover well, and those that don’t usually lack regeneration, the data focuses on animals that recovered good locomotor function.

**FIGURE 2:**
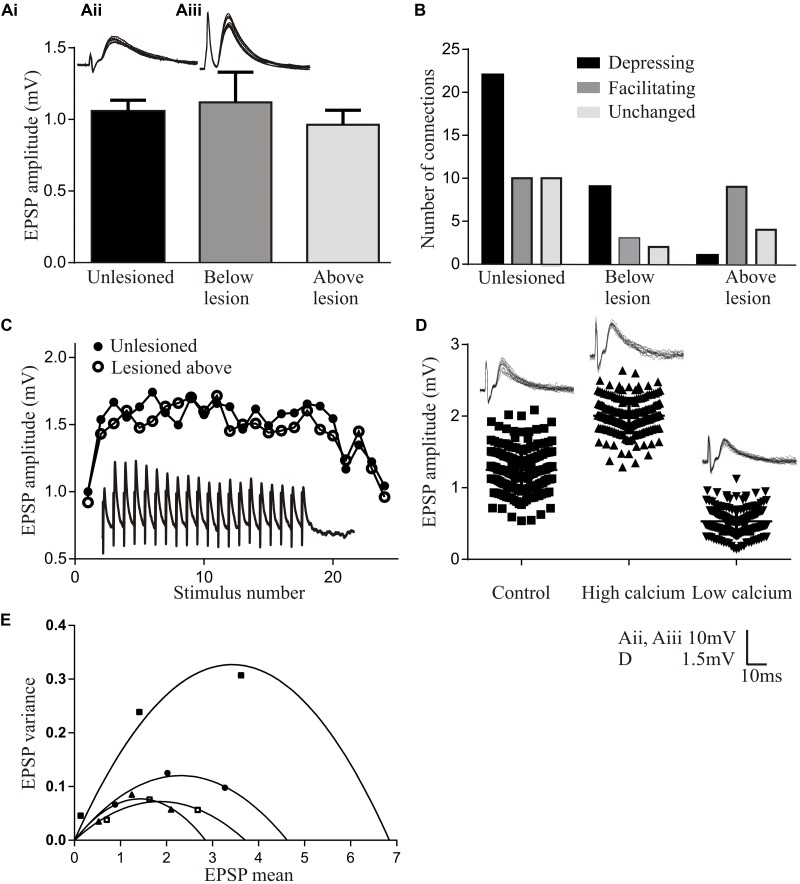
Properties of regenerated synapses. **(Ai)** The initial EPSP amplitude from reticulospinal axons in unlesioned and lesioned spinal cords. The inset shows the largest response seen in an unlesioned spinal cord **(Aii)**, and the occasional very large inputs seen in lesioned animals **(Aiii)**. **(B)** Proportions of connections showing different forms of activity-dependent plasticity in unlesioned and lesioned spinal cords. **(C)** Graph comparing facilitation in unlesioned spinal cords and from above the lesion site. The inset shows facilitation above the lesion. **(D)** The effect of high and low Ca^2+^ on the properties of reticulospinal EPSPS. **(E)** Variance-mean plots in high and low calcium for four connections.

The basic properties of reticulospinal inputs, the amplitude (**Figure 2Ai**), rise-time and half-width, did not differ significantly in lesioned and unlesioned animals. This suggests against any compensation at individual synapses for the reduced innervation of the sub-lesion spinal cord that will occur given that regeneration is never complete (see Frank, 2014). However, we occasionally find responses that are much larger than the largest responses in unlesioned animals (>10 mV; Cooke and Parker, 2009) (**Figures 2Aii,Aiii**), suggesting increased variability after lesioning. In unlesioned animals most connections depressed over spike trains (**Figure 2B**). The proportion of connections showing different forms of plasticity was the same below the lesion site as in unlesioned animals, but above the lesion site connections usually facilitated (**Figures 2B,C**). While this analysis is on lateral tract axons, these features were also seen for putative Muller axons (Cooke and Parker, 2009; Parker, unpublished observations).

Regenerated Muller axons in the ventromedial region of the spinal cord make fewer synapses below the lesion site, contain fewer vesicles, and have smaller active zones than comparable synapses from unlesioned animals (Oliphint et al., 2010). These ultrastructural differences make it surprising that regenerated inputs matched those in unlesioned animals (Cooke and Parker, 2009), and suggest that some adaptation is needed for regenerated synapses to maintain the same output. This was examined using a variance-mean analysis (see Clements and Silver, 2000 for details), which examines excitatory postsynaptic potentials (EPSPs) in normal, high, and low calcium Ringer. Plotting the EPSP variance against the mean under these conditions results in a parabolic relationship (unless the release probability is low (<0.3) when the relationship is linear). The parabolic fit allows synaptic parameters to be estimated (**Figures 2D,E**): the initial slope represents Q (the quantal amplitude, the postsynaptic response to a single vehicle), the width represents N (the number of release sites), and the degree of curvature represents Pr (the transmitter release probability). These analyses were performed in the larger medial column Muller axons, firstly to relate functional properties to the ultrastructural features in Oliphint et al. (2010), and secondly because they allow the stable recordings needed for the multiple Ringer changes needed for this analysis.

In regenerated axons below the lesion site N was generally reduced, consistent with the sparser anatomical connectivity (Oliphint et al., 2010); Pr was generally unchanged, consistent with a lack of difference in activity-dependent plasticity (see above); but Q was consistently increased, suggesting a change in postsynaptic responsiveness. Analyses of connections above the lesion site showed a linear relationship between the variance and mean indicative of a low release probability (Pr < 0.3; Clements and Silver, 2000). This prevented estimates of N and Pr. However, Q could be determined, and was consistently increased.

While these analyses are not definitive, they suggest lesion-induced changes of reticulospinal synapses that differ above and below the lesion site. The increased Q below the lesion site could scale regenerated inputs so that they match unlesioned synapses despite the sparser connectivity. The postsynaptic increase in Q but reduced Pr above the lesion site allows connections to facilitate without the reduction of the initial input that would occur with a reduction of Pr alone, resulting in a net increase in connection strength above the lesion site (see Bevan and Parker, 2004). A reduction of transmitter release associated with a reduced Pr is also energetically favorable (Howarth et al., 2012), a potentially important consideration in injured nervous systems.

While this analysis focuses on animals that recovered well, connections have been examined below the lesion site in animals that failed to recover despite regeneration. The sample size is small (*n* = 4) making the data very preliminary. These connections had initial inputs that were markedly smaller than unlesioned connections (<0.3 mV) and tended to depress strongly, to the extent that the connections failed (connections were monosynaptic as they persisted in high divalent Ringer). This suggests a potential failure of the postsynaptic scaling of the connection (increase in Q), while compromised function over repetitive stimulation could reflect a failure of activity-dependent replenishment mechanisms (Parker, 2000).

A feature to note from these analyses is the marked variability of connections (see **Figure 2E** for an example of various-mean plots from four individual axons). Averaging these responses to give mean values does not seem appropriate. Synapses vary in unlesioned animals (Brodin et al., 1994), but this seems to be increased after lesioning (Cooke and Parker, 2009). Larger sample sizes are needed to characterize this variability and the functional properties of regenerated synapses (Parker, 2003a).

## Changes in Spinal Cord Networks

Even with the highest levels of regeneration (∼70%; McClellan, 1994), the apparent absence of a change in the amplitude of individual regenerated synapses means that the descending drive to the spinal cord will be reduced. This can only be avoided if there was redundancy or degeneracy in the descending input in unlesioned animals, or if there was some adaptation below the lesion site that allowed the same output to be generated from a reduced descending input. Selzer (1978) suggested that functional recovery in lamprey would reflect a combination of regeneration and network plasticity, but the latter has subsequently received little attention. We have thus examined changes in the locomotor network above and below the lesion site.

**FIGURE 3:**
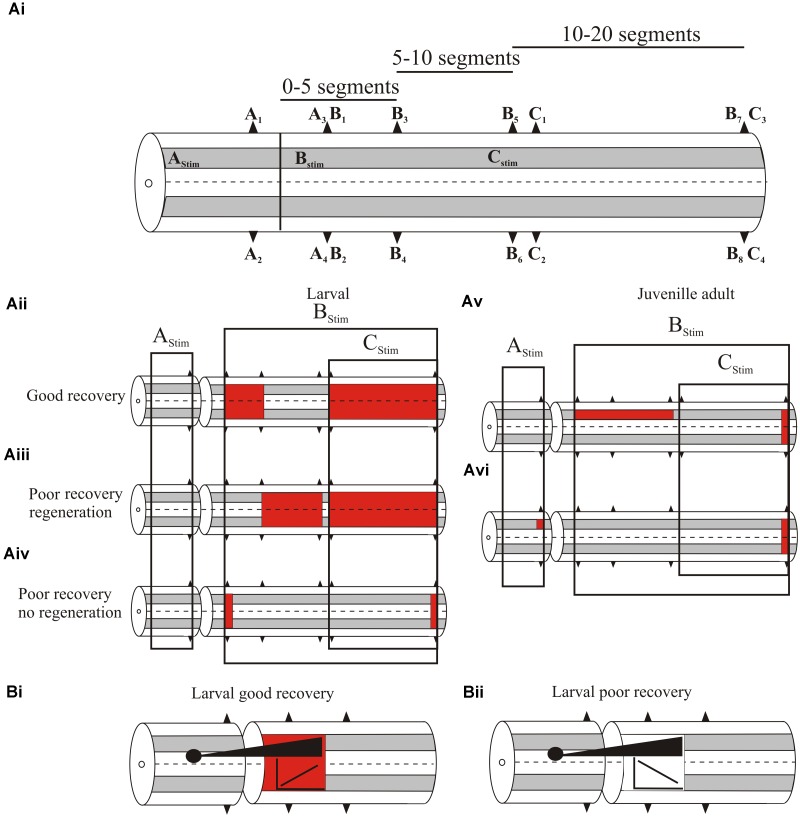
**(Ai)** Experimental approach to examine changes in spinal cord excitability. A_1_–C_4_ are ventral root locations along the body relative to a lesion site (or where the lesion would be in an unlesioned spinal cord). A_1_–A_2_ are ipsilateral or contralateral ventral root responses recorded one segment above the lesion in response to stimulation three segments above the lesion (A_Stim_); A_3_ and A_4_ are ipsilateral and contralateral responses, respectively, evoked by A_Stim_ two segments below the lesion site. B_1_–B_8_ are ventral root locations 2–20 segments below the lesion site (B_1/2_, 2 segments below; B_3/4_, 5 segments below; B_5/6_, 10 segments below B_7/8_, 20 segments below) ipsilateral to stimulation one segment below the lesion (B_Stim_). C_1-4_ are ventral root locations 10–20 segments below the lesion site (C_1/2_, 11 segments below; C_3/4_, 20 segments below) in response to stimulation 10 segments below the lesion (C_Stim_). **(Aii)** Changes in excitability in larvae that showed good recovery. The colored squares represent increased excitability for stimulation in the regions indicated by the boxes. **(Aiii)** Excitability changes in larvae that showed poor recovery and regeneration, and **(Aiv)** in larvae that showed poor recovery and no regeneration. **(Av)** Excitability changes in juvenile adults that showed poor recovery. **(Avi)** Excitability changes in juvenile adults that showed good recovery. **(Bi)** The relationship between the extent of regeneration and the excitability below the lesion site. With good recovery sub-lesion excitability increased as regeneration increased, but in poor recovery excitability decreased as regeneration increased **(Bii)**.

We initially examined global levels of spinal cord excitability by recording extracellular activity from ventral roots or the surface of the spinal cord (both responses are generally similar) in response to spinal cord stimulation at different supra- and sub-lesion sites (or comparable regions in the unlesioned spinal cord; **Figure 3Ai**; Cooke and Parker, 2009; Hoffman and Parker, 2011; Parker, unpublished data). In larvae, with good recovery excitability was increased locally (up to five segments) below, but was not altered above the lesion site (**Figure 3Aii**; Cooke and Parker, 2009). Excitability was also increased distal to the lesion site (11–20 segments below the lesion in response to stimulation 10 segments below; **Figure 3Aii**). Effects differed in poorly recovered animals, depending on whether regeneration occurred: in poor recovery with regeneration excitability was not increased locally only distally below the lesion site (**Figure 3Aiii**), but without regeneration excitability was increased immediately below the lesion site but distal effects were reduced (**Figure 3iv**). This suggests several aspects: firstly, increased excitability locally (1–5 segments) below the lesion site is needed for good recovery; secondly, excitability changes occur diffusely below the lesion site, (see Grasso et al., 2004 for a similar effect in the human spinal cord); and finally, regenerated inputs influence the excitability changes.

While increased excitability was associated with recovery, there was marked variability in responses between animals, and overall the excitability changes did not correlate statistically significantly with the degree of recovery. Several factors could underlie this variability: the magnitude of extracellular signals could have differed due to electrode location in different animals; and secondly, descending inputs were activated using relatively crude stimulation that did not address regional (various descending or ascending pathways, propriospinal or reticulospinal inputs) or other divisions (e.g., transmitter content) that may better reveal a link between the excitability changes and degree of regeneration. Alternatively, rather than being “noise” associated with these effects, variability may be a signal that allows the spinal cord to sample a range of options to optimize recovery (Ashby, 1958), or it reflects the need for spinal cord excitability to be adapted to the varying influence of sensory and regenerated inputs.

Juvenile adults also showed changes in excitability. In contrast to larvae these were significantly related to different degrees of recovery. Animals that failed to recover had significantly increased excitability ipsilateral to the stimulation site up to 10 segments below the lesion (**Figure 3Av**), and a significant reduction of activity when stimulating across the lesion site, indicative of a lack of functional regeneration. In animals that showed good recovery, regeneration had occurred and there was a significant increase in excitability ipsilateral to stimulation above the lesion site (**Figure 3Avi**). However, below the lesion site excitability did not differ significantly to unlesioned animals. This suggests that in adults as regeneration occurs sub-lesion excitability falls back to match that in unlesioned animals. However, this restoration was associated with changes in functional properties and spinal cord organization (see below).

A significant feature of this analysis was the interaction of regenerated inputs with sub-lesion networks in larvae. As outlined above, the relationship between regeneration and recovery is inconsistent (Steward et al., 2012). Rather than regeneration *per se*, recovery may depend on how regenerated inputs interact with sub-lesion networks. When animals showed good recovery there was a positive correlation between the extent of regeneration (assessed from the A3/A4 responses) and the magnitude of the excitability changes below the lesion site (B1–B4 responses; **Figure 3Bi**). However, animals that failed to recover showed negative correlations between the degree of regeneration and excitability (**Figure 3Bii**; Hoffman and Parker, 2011). If the changes in sub-lesion excitability are compensations for the loss of descending excitation, regeneration should lead to adjustments of excitability. In poor recovery this adjustment may be excessive, leading to the negative correlation shown here. This interaction needs further study, but it supports the idea that recovery is more than regeneration.

## Below Lesion Cellular Changes

Understanding the changes in spinal cord networks and the interactions with regenerated inputs requires understanding the cellular mechanisms of the lesion-induced changes. These have been examined by recording from spinal cord neurons above and below the lesion site. The analysis has focused on motor neurons and their inputs, as these are easier to record from. However, as effects cannot be generalized between neurons or synapses (Parker, 2006) analyses of locomotor network interneurons has started.

**FIGURE 4:**
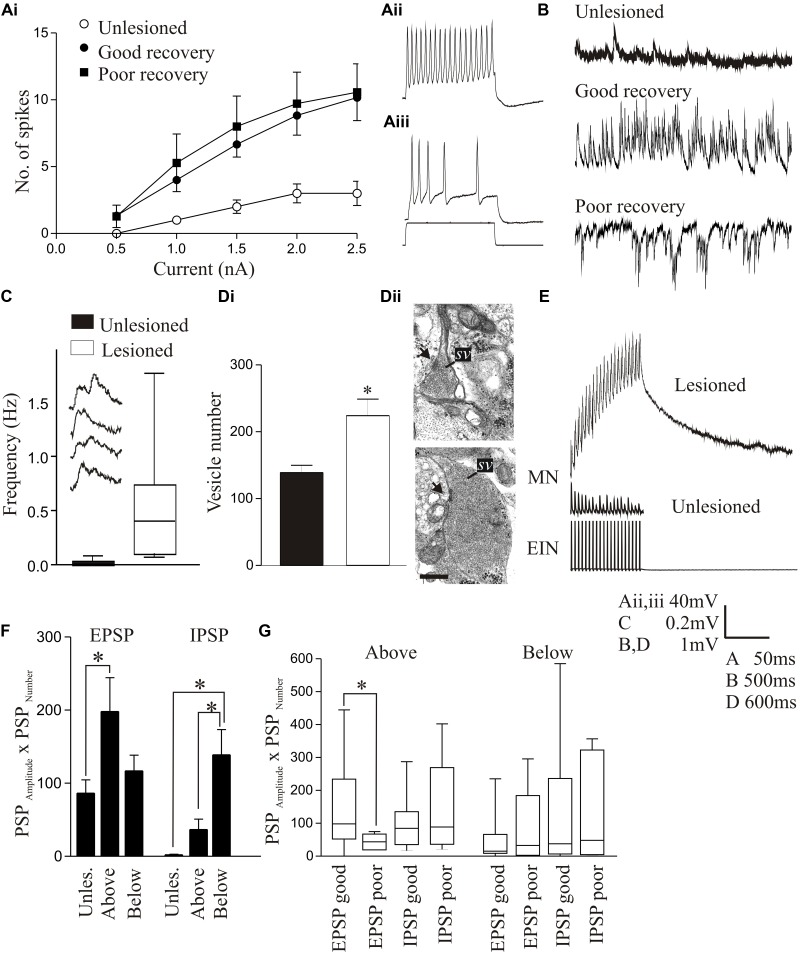
Lesion-induced changes in cellular properties. **(Ai)** Graph showing changes in excitability in unlesioned larvae, and in lesioned larvae showing good and poor recovery. Traces show excitability changes in a lesioned **(Aii)** and unlesioned animal **(Aiii)**. **(B)** Spontaneous synaptic inputs in an unlesioned spinal cord, and below the lesion site in animals that showed good or poor recovery. **(C)** Graph and traces showing “double” spontaneous miniature EPSPs. **(Di)** Graph showing the number of vesicles in synaptic terminals in an unlesioned and a lesioned spinal cord. **(Dii)** Electron micrographs showing examples of putative glutamatergic synapses in an unlesioned (top) and a lesioned spinal cord (below). **(E)** Example of an excitatory interneuron to motor neuron connection in an unlesioned animal and the slow depolarization that occurs at the same type of connection in a lesioned animal. **(F)** Changes in spontaneous synaptic inputs above and below the lesion site in juvenile adult animals. **(G)** Changes in excitatory and inhibitory synaptic inputs above and below the lesion site in juvenile animals that recovered well or poorly. Data is presented as a boxplot to indicate the variability. Data from Cooke and Parker (2009) and Becker and Parker (2015). No permission is required to reproduce this material. ^∗^Indicates statistical significance at *p* < 0.05.

In larvae, the resting membrane potential, input resistance, and excitability in response to depolarizing current pulses (including the appearance of plateau potentials) were all significantly increased in motor neurons below the lesion site compared to unlesioned animals (**Figures 4Ai–Aiii**; Cooke and Parker, 2009). These changes occurred in animals that recovered well or poorly, suggesting that they don’t directly determine the degree of recovery. There was a significant increase in the spontaneous EPSP amplitude and frequency below the lesion site with good and poor recovery, but the IPSP amplitude and frequency were significantly increased with poor recovery (**Figure 4B**). As a result, the synaptic excitation:inhibition ratio was increased in animals that recovered well compared to unlesioned animals (9.84 and 2.45, respectively), but was reduced in animals that failed to recover (1.36; Cooke and Parker, 2009). This provides a link to a cellular mechanism for the changes in spinal cord excitability outlined above. Similar cellular and synaptic effects occur in the glutamatergic excitatory interneurons (EIN), which are identified by their ability to make monosynaptic connections onto motor neurons and other network interneurons (Buchanan, 2001), including the development of plateau potentials and marked increase in spontaneous excitatory synaptic inputs, although the sample size of recordings from these cells is still relatively small (Cooke and Parker, 2009; Parker, unpublished observations). These changes may be needed to compensate for the reduced descending excitation, but also for a reduction in intraspinal glutamatergic interneurons (Fernández-López et al., 2016).

Synaptic effects below the lesion site were examined in more detail using TTX-resistant miniature EPSPs (mEPSPs) to remove the influence of changes in cellular excitability. The amplitude, frequency, and half-width of mEPSPs were all increased, and there was an increase in summated or “double” mEPSPs (**Figure 4C**). These effects could reflect presynaptic and postsynaptic changes. An ultrastructural analysis of synaptic terminals from putative network interneurons showed that in lesioned animals the total and docked vesicle numbers were significantly greater (**Figures 4Di,Dii**) and the vesicle diameter and synaptic gap significantly reduced at asymmetric (putative excitatory) synapses. However, there was a reduction in the post-synaptic density length at symmetric (putative inhibitory) synapses (Cooke and Parker, 2009). These effects were only studied in animals that showed good recovery, and thus we do not yet know whether they discriminate between recovery stages.

Despite evidence for changes in excitatory synaptic ultrastructure after lesioning, as with regenerated axon synapses there were no significant differences in the amplitude, rise-time or half-width of single EPSPs evoked by EINs to motor neurons (Cooke and Parker, 2009). However, during spike trains a very pronounced slow depolarization developed that was not seen in unlesioned animals (**Figure 4E**). This was significantly larger in animals that recovered well than those that recovered poorly where it was small or absent, suggesting another potential factor in good recovery. The mechanism underlying this effect is unknown. It does not seem to reflect an L-type calcium, persistent sodium, or NMDA conductance (see Li et al., 2004a), increased EPSP summation due to changes in half-width, or the recruitment of polysynaptic inputs (Cooke and Parker, 2009; Becker and Parker, 2015). One possibility is that it reflects “synaptic drag” (Martin and Pilar, 1964), an increase in asynchronous vesicle release that can cause slowly developing depolarizations with repetitive stimulation (Hjelmstad, 2005; Iremonger and Bains, 2007). This is supported by the double mEPSPs in lesioned animals (**Figure 4C**), which could be facilitated by the larger glutamatergic vesicle pool. We tried to mimic the effect in unlesioned animals using strontium to increase spontaneous release and *N*-ethylmaleimide to increase the number of docked vesicles (Lonart and Sudhof, 2000), but have failed to evoke a slow depolarization (Parker, unpublished observations). While this offers no support for vesicle “drag,” we cannot yet claim to have mimicked the properties of synapses in lesioned animals.

In contrast to larvae, juvenile adult animals showed no statistically significant differences in motor neuron cellular properties after lesioning [resting potential, input resistance, slow afterhyperpolarization (sAHP), excitability; Becker and Parker, 2015], but there was an increase in spontaneous excitatory and inhibitory synaptic inputs (**Figure 4F**). Unlike larvae, where increased inhibition was associated with poor recovery, in juvenile animals increased inhibition also occurred in good recovery (**Figures 4F,G**; Becker and Parker, 2015). This may be a necessary requirement to deal with developmental differences in spinal cord circuitry: inhibition is reduced in juveniles compared to larvae (Parker and Gilbey, 2007), which could make juveniles more susceptible to runaway excitation after SCI. A lack of appropriate inhibition is associated with various dysfunctions [e.g., dystonia (Quartarone et al., 2008); neuropathic pain (Kuner, 2010)]. This suggests that activation of sub-lesion networks is not simply about increasing excitation, but about ensuring the appropriate balance between excitation and inhibition (Berg et al., 2007).

## Above Lesion Changes

The defects in locomotor activity (**Figure 1Diii**) and changes in excitability (**Figure 3Avi**) suggest that there are changes above the lesion site. This may reflect the degeneration of damaged neurons above the lesion site. In addition, there are ascending propriospinal inputs to the spinal cord and brainstem that will be affected by a lesion (see Armstrong et al., 2003 for lamprey). This could result in diaschisis above the lesion site, either by the removal of ascending inputs or by changes in their properties after regeneration. Changes above the lesion (e.g., increased excitation; **Figure 3Avi**) may also be programmed directly to increase propriospinal signals across the lesion site to compensate for the reduction of descending brainstem inputs (Courtine et al., 2008). We have thus also examined cellular changes above the lesion site, so far only in juvenile animals (Becker and Parker, 2015).

**FIGURE 5:**
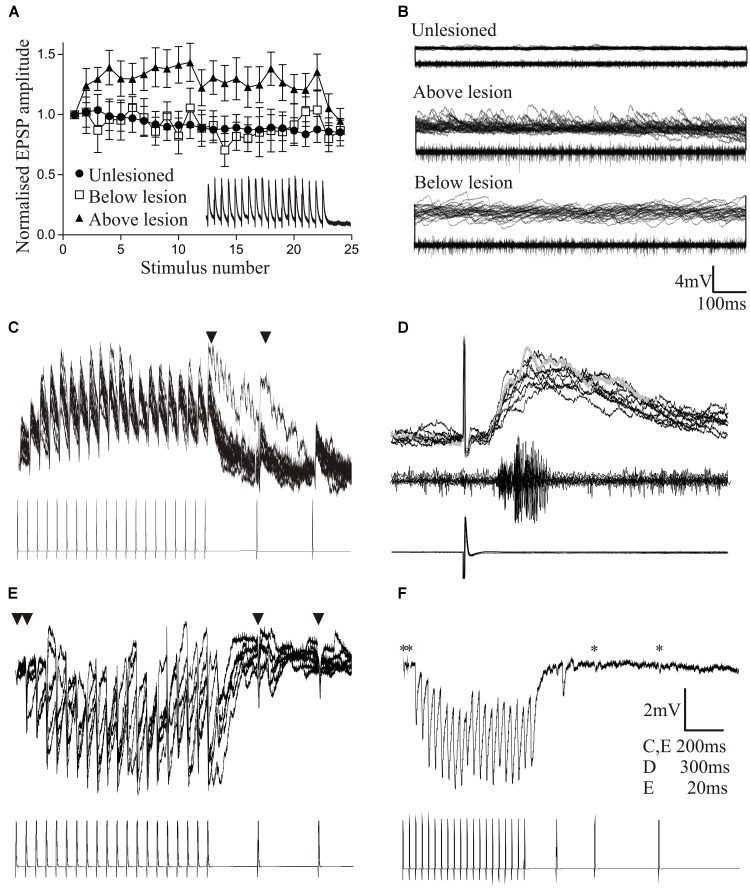
Changes in reticulospinal-evoked synaptic inputs to motor neurons in unlesioned juvenile animals, and lesioned animals above and below the lesion site. **(A)** Note the facilitation above, but depression below the lesion site and in unlesioned animals. **(B)** Spontaneous synaptic inputs above and below the lesion site in juvenile adult animals. **(C,D)** Evidence for polysynaptic excitatory synaptic interactions (indicated by ^∗^) above the lesion site. **(E,F)** Polysynaptic inhibition below the lesion site in juvenile adult animals (in this case ^∗^ refers to inputs that failed to evoke an input, IPSPs only developing during the spike train as a result of activation of the feedforward inhibitory pathway). Data from Becker and Parker (2015). No permission is required to reproduce this material.

Cellular properties (resting potential, input resistance, sAHP, excitability) and the slow synaptic depolarization did not differ significantly above and below the lesion site to the properties in unlesioned animals (Becker and Parker, 2015). However, synaptic effects did differ. As outlined above, reticulospinal inputs significantly facilitated above but depressed below the lesion site (**Figures 2C**, **5A**). The frequency of spontaneous excitatory and inhibitory synaptic inputs was increased above and below the lesion site compared to unlesioned animals, but spontaneous inhibitory inputs were greater below and excitatory inputs greater above the lesion (**Figures 4F**, **5B**). The increased excitatory input above the lesion was lacking in animals that failed to recover (**Figure 4G**), again suggesting a potential role in good recovery, but the increased inhibition below the lesion site occurred irrespective of the degree of recovery. The changes in supra-lesion excitation were supported by the increased feedforward excitatory interactions above the lesion site seen in paired recordings from EINs and motor neurons (**Figures 5C,D**, and an increased connection probability between EINs (from a very sparse unlesioned probability of <0.1 (Jia and Parker, 2016) to a probability of 0.5; but *n* = 4 connections). However, feedforward inhibition was increased below the lesion (**Figures 5E,F**). These effects suggest a potential difference in the reorganization of sub and supra-lesion locomotor networks (Becker and Parker, 2015),

To summarize, there are significant changes in locomotor network cellular and synaptic properties after lesioning, that are specific to the developmental stage, degree of recovery, and location relative to the lesion site. There is also evidence for the reorganization of locomotor networks above and below the lesion site, shown by the increased feedforward excitation and inhibition, respectively. These changes support the claim that the lesioned spinal cord is functionally “new” (Edgerton et al., 2001). Some of the changes correlated significantly with recovery (e.g., sub and supra-lesion excitability, excitation:inhibition ratio, interaction of regenerated inputs with sub-lesion excitability, slow synaptic depolarization, role of inhibition), and could suggest potential compensations for the reduced descending drive to motor neurons and EINs. As animals that showed poor recovery always lacked regeneration these changes alone are presumably insufficient for recovery. Lurie and Selzer (1991) showed that re-transection abolished recovered locomotor function in lamprey (*n* = 2), suggesting that regeneration was necessary. Interestingly, recovery was faster after the second transection (Wood and Cohen, 1981; Lurie and Selzer, 1991), possibly reflecting the prior development of the functional changes (see Courtine et al., 2008 and Frigon et al., 2009 for similar effects in mammals). Re-transection also only shows that regeneration is necessary, not that it is sufficient for recovery, and does not rule out some role for the sub-lesion changes. A feature is again variability (see **Figures 4F,G**). This may be “noise” (e.g., differences between animals, or poorly defined neuronal populations; Parker, 2003a), but may again be a signal that allows the spinal cord to explore a range of options to optimize recovery.

The changes in cellular properties in larval EINs and motor neurons suggest a switch from synaptic to cellular driven excitability that may allow these cells to be activated by reduced synaptic inputs. This may be advantageous: the main energy cost in the nervous system is for synaptic transmission (Howarth et al., 2012), and as sources of energy may be compromised after injury or used elsewhere (e.g., in repair), this economy might be beneficial. However, this switch does not seem to occur in juvenile animals, where synaptic effects dominate. The reasons for this are unknown, but may reflect differing network requirements. Juveniles actively swim whereas larvae are passive and swim only when disturbed. This is associated with differences in the locomotor network (Parker and Gilbey, 2007), and the more active juveniles may need a network where synaptic co-ordination rather than intrinsic cellular properties are more important for ensuring optimal activity. This needs further work, but the differences in these systems offer the chance to examine how systems with differing initial properties respond to perturbation.

Inhibitory inputs, most likely from the glycinergic small ipsilateral inhibitory interneurons (SiINs) that are identified by their ability to make monosynaptic connections onto motor neurons (Buchanan, 2001) and the EINs (Parker, 2003b), could have a key role. These have opposite influences in larvae and adults on the degree of recovery. While these neurons have been removed from some network schemes (Grillner, 2003), they have criteria consistent with a network role (Buchanan and Grillner, 1988), and can modify the effects of EIN-driven network activity (Jia and Parker, 2016). This modifying effect may also underlie their differing influence on recovery in larvae and juveniles, making consideration of inhibitory control rather, than excitation, a consideration in recovery strategies.

## Sensory Changes

**FIGURE 6:**
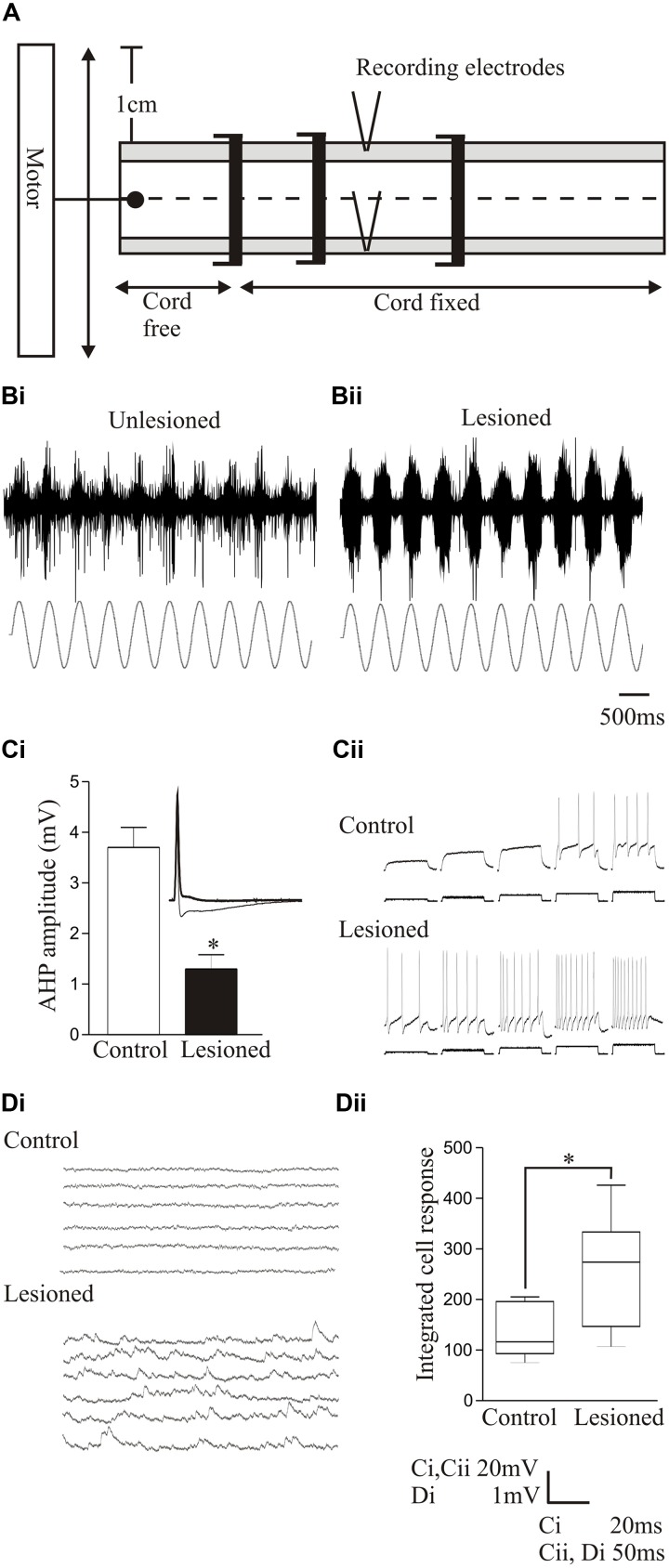
Changes in sensory feedback after lesioning. **(A)** Diagram showing the experimental procedure for evoking and monitoring proprioceptive feedback to the spinal cord. The spinal cord is fixed except at a free end that is attached to a computer-driven motor that imposes sinusoidal movements of the cord. Movement-dependent activity is recorded from the lateral tract where edge cell axons run. Stretch-evoked responses to a 1 Hz bending command in an unlesioned **(Bi)** and lesioned spinal cord **(Bii)**. **(Ci)** Graph showing the significant reduction of the post-spike slow afterhyperpolarization (sAHP) in an edge cell in a lesioned spinal cord. The inset shows an edge cell action potential in an unlesioned and lesioned spinal cord (thick line). **(Cii)** Traces showing the increase in edge cell excitability in response to depolarizing current injection in the unlesioned and lesioned spinal cord. **(Di)** Traces showing spontaneous synaptic inputs in an edge cell in an unlesioned and lesioned spinal cord. **(Dii)** Graph showing the significant increase in the integrated spontaneous synaptic input in edge cells after lesioning. Data from Hoffman and Parker (2011). Permission granted to reproduce. ^∗^Indicates statistical significance at *p* < 0.05.

Proprioceptive inputs can be examined simply in the lamprey as the proprioceptive edge cells are located on the lateral margin of the spinal cord (Rovainen, 1974). They serve a similar function as mammalian muscle spindles, but monitor stretch of the spinal cord rather than muscles (Vinay et al., 1996). Edge cell axons project to the ipsilateral and contralateral side of the spinal cord where they make excitatory and inhibitory connections, respectively (Di Prisco et al., 1990). The spinal location of these cells allows sensory inputs to be examined by imposing sinusoidal movements onto the isolated spinal cord while monitoring the movement-evoked activity from the lateral tracts where edge cell axons run (**Figure 6A**; McClellan and Sigvardt, 1988).

After recovery from lesioning, sensory feedback was potentiated and adaptation reduced in response to bending-evoked stretch of the spinal cord (**Figures 6Bi,Bii**; Hoffman and Parker, 2011; Svensson et al., 2013). Intracellular recordings showed that lesioning depolarized the edge cell resting potential, increased the input resistance, and reduced the sAHP after an action potential (**Figure 6Ci**), effects associated with an increase in the excitability in response to current injection (**Figure 6Cii**). There also seems to be an interaction of sensory systems with the locomotor network, as after lesioning there was an increase in spontaneous synaptic inputs in the edge cells (**Figures 6Di,Dii**; Hoffman and Parker, 2011). This presumably reflects locomotor network inputs that regulate edge cell responses (Vinay et al., 1996), and again illustrates the interactions between components rather than the focus on single properties. The sensory potentiation may be a direct adaptive response to the reduced excitability below the lesion site caused by the reduced descending excitation, or a secondary effect triggered by the need to monitor and adjust the altered locomotor network output. The potentiation seems to be specific to proprioceptive inputs, as the somatosensory dorsal cells are not significantly affected by lesioning (Parker, unpublished data).

While the sensory changes should have had some positive or negative influence on locomotion, there was no significant correlation between the sensory potentiation and the degree of locomotor recovery (Hoffman and Parker, 2011). There was also no significant correlation between the sensory potentiation and the extent of regeneration, suggesting that while the effect is triggered by the lesion, it is not regulated by regenerated inputs. It is possible that a threshold level of descending input is needed to reverse the sensory potentiation that was not reached in our experiments, or that the sensory potentiation reverses at later recovery times. Alternatively, it may reflect a secondary adaptation to the changing locomotor network. It has been argued that sensory input is not needed for functional recovery (McClellan, 1990) because fictive locomotion can be evoked in the isolated spinal cord. This is not good evidence, but McClellan (1990) also examined the involvement of sensory inputs by re-transecting the spinal cord after recovery or by removing a 2–5 mm region of the spinal cord. Both approaches abolished activity below the lesion site even though body movements spread mechanically below the lesion site. This suggests that sensory inputs were unable to detect or relay sub-lesion movements to locomotor networks to propagate activity below the lesion site. However, this does not rule out some contribution of sensory feedback in recovery.

Various types of locomotor activity can improve motor function after SCI (Wernig et al., 1995; D’Amico et al., 2014). Locomotor recovery in lamprey does not seem to be activity-dependent, as restraining animals in plastic tubes to prevent movement did not abolish locomotor recovery or the potentiation of sensory responses (Hoffman and Parker unpublished data; see also Cohen et al., 1988). While the restraint was not total, movement was markedly reduced and some effect would have been expected if movement was needed for recovery.

Changes in sensory inputs have been implicated in the development of spasticity (Li et al., 2004b; Mukherjee and Chakravarty, 2010). They are also targeted in treadmill training to enhance motor function below a lesion site (Smith and Knikou, 2016), and have been implicated in locomotor recovery following electrical or pharmacological stimulation of the sub-lesion spinal cord (Lavrov et al., 2008). While the lack of correlation between the sensory potentiation and degree of recovery in lamprey seems surprising, the relationship between sensory changes and function after SCI generally seems complex (see Smith and Knikou, 2016). For example, there was a lack of correlation between stretch reflex-activated inputs (analogous to edge cell inputs in lamprey) and stepping after human SCI (Dietz et al., 2002), and a lack of correlation between H reflex changes and behavioral scores assessed from hind limb function in rat (Lee et al., 2005). Lee et al. (2005) also showed a correlation between spared white matter after contusion injury and hindlimb function, but no correlation between the degree of spared white matter and H reflex changes, mimicking the lack of correlation between regeneration and the sensory changes that we saw. Thus, while changes in sensory systems occur routinely after SCI, their influence is complicated (Morawietz and Moffat, 2013; Smith and Knikou, 2016), and suggests a role that involves interactions with other motor system components.

## Neuromodulation

The existence of spinal cord locomotor networks that can be activated pharmacologically has led to the search for pharmacological approaches for activating the sub-lesion spinal cord. Despite a vast literature on drug effects (Rossignol et al., 2001; Tillakaratne et al., 2002; Barbeau and Norman, 2003; Parker, 2005), there is still little insight into what might constitute an optimal pharmacological treatment for SCI. This is complicated by the diversity of neuron and synapse-specific effects that make a complete functional description of any neuromodulator difficult; by state-dependent influences that can alter modulatory effects; and by interactions between modulatory systems (see for example, Yang and Faber, 1991; Katz and Frost, 1995; Harris-Warrick et al., 1998; Katz and Edwards, 1999; Svensson et al., 2001; Sakurai and Katz, 2003; Brezina, 2010; Harris-Warrick and Johnson, 2010; Zhao et al., 2011; Parker, 2015). We have started to examine modulatory effects after lesioning in lamprey (Svensson et al., 2013; Becker and Parker, 2015; McClelland and Parker, 2017) as a parallel to studies of anatomical changes in transmitter systems (Cohen et al., 2005; Cornide-Petronio et al., 2014; Fernández-López et al., 2014, 2015).

**FIGURE 7:**
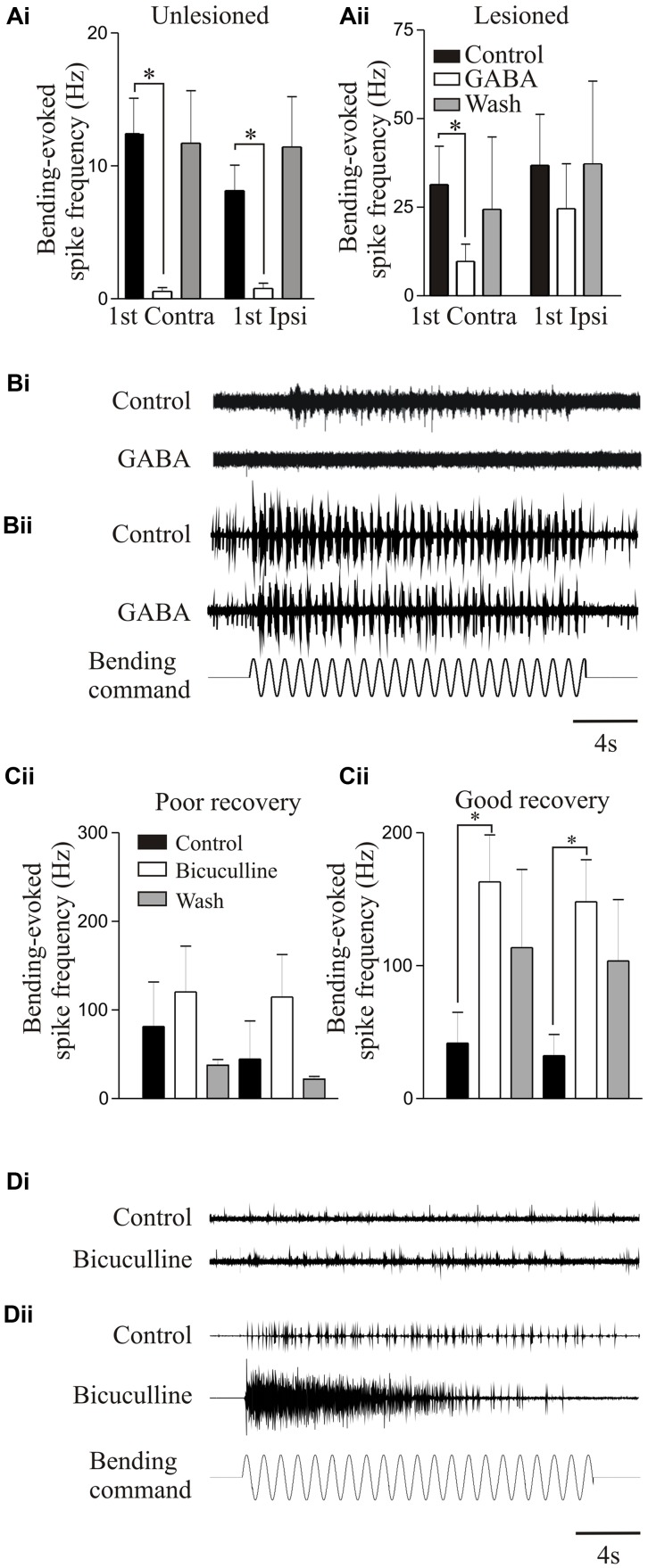
Modulation of proprioceptive activity. **(Ai,Aii)** The effect of GABA on bending-evoked activity recorded from the lateral margin of the spinal cord. Notice that GABA effects are weaker in lesioned animals. Traces showing proprioceptive activity in an unlesioned **(Bi)** and a lesioned animal **(Bii)**. **(Ci,Cii)** The effects of bicuculline on bending-evoked activity depended on the degree of recovery: note that bicuculline effects are only significant in lesioned animals that showed good recovery. Traces showing the effects of bicuculline on bending-evoked activity in animals that showed poor **(Di)** and good recovery **(Dii)**. Data from Svensson et al. (2013). Permission granted to reproduce. ^∗^Indicates statistical significance at *p* < 0.05.

Proprioceptive inputs offered a convenient system for examining modulatory effects as they can be examined relatively easily, and receive modulatory inputs from cells that co-localize GABA and somatostatin (Christenson et al., 1991). Exogenously applied GABA significantly reduced proprioceptive responses in unlesioned animals (**Figures 7Ai,Bi**). But in lesioned animals, where proprioceptive activity was potentiated (see above), GABA was less effective (**Figures 7Aii,Bii**; Svensson et al., 2013). Reduced GABA inhibition could have accounted for the potentiated proprioceptive response. However, blocking endogenous GABA with bicuculline significantly potentiated proprioceptive responses in lesioned, but not unlesioned, animals (**Figures 7Ci,Cii**; Svensson et al., 2013), suggesting that there was increased tonic GABAergic inhibition after lesioning. This may be an example of the need for potentiated excitatory effects to be balanced by increased inhibition (see above, Becker and Parker, 2015).

Bicuculline could cause long-lasting sensory discharges, suggesting that unregulated proprioceptive feedback would disrupt locomotion. Bicuculline can improve locomotor function after SCI when the lesion was made in adult cats and resulting recovery was poor; but disrupted activity when the lesion was made in kittens that subsequently recovered good locomotor function, suggesting that increased GABAergic inhibition was needed for recovery (Robinson and Goldberger, 1986a,b). This was mimicked in lamprey: bicuculline only potentiated proprioceptive responses when animals recovered good locomotor function, suggesting increased GABA levels occur with good recovery (**Figures 7Ci–Dii**).

Somatostatin, which co-localizes with GABA (Christenson et al., 1991), did not affect proprioceptive responses on its own in lesioned or unlesioned animals. However, in lesioned animals, it further reduced the effects of GABA (Svensson et al., 2013), suggesting a lesion-induced metamodulatory influence that regulates GABA effects.

5-HT is arguably the dominant transmitter system studied after SCI. 5-HT has significant effects on motor outputs and sensory processing in the unlesioned spinal cord (Schmidt and Jordan, 2000), and there is evidence that 5-HT receptor levels (Giroux et al., 1999; Otoshi et al., 2009) and 5-HT receptor agonists can influence locomotion after injury (Giménez y Ribotta et al., 1998; Hains et al., 2001; Hochman et al., 2001; Antri et al., 2003; Nothias et al., 2005; Murray et al., 2010; D’Amico et al., 2013; Barreiro-Iglesias et al., 2015; see Gackière and Vinay, 2014, for a recent review). However, the mechanisms underlying any improvements, which should be targeted to improve recovery, are unclear. We have thus started to examine 5-HT effects after lesioning.

**FIGURE 8:**
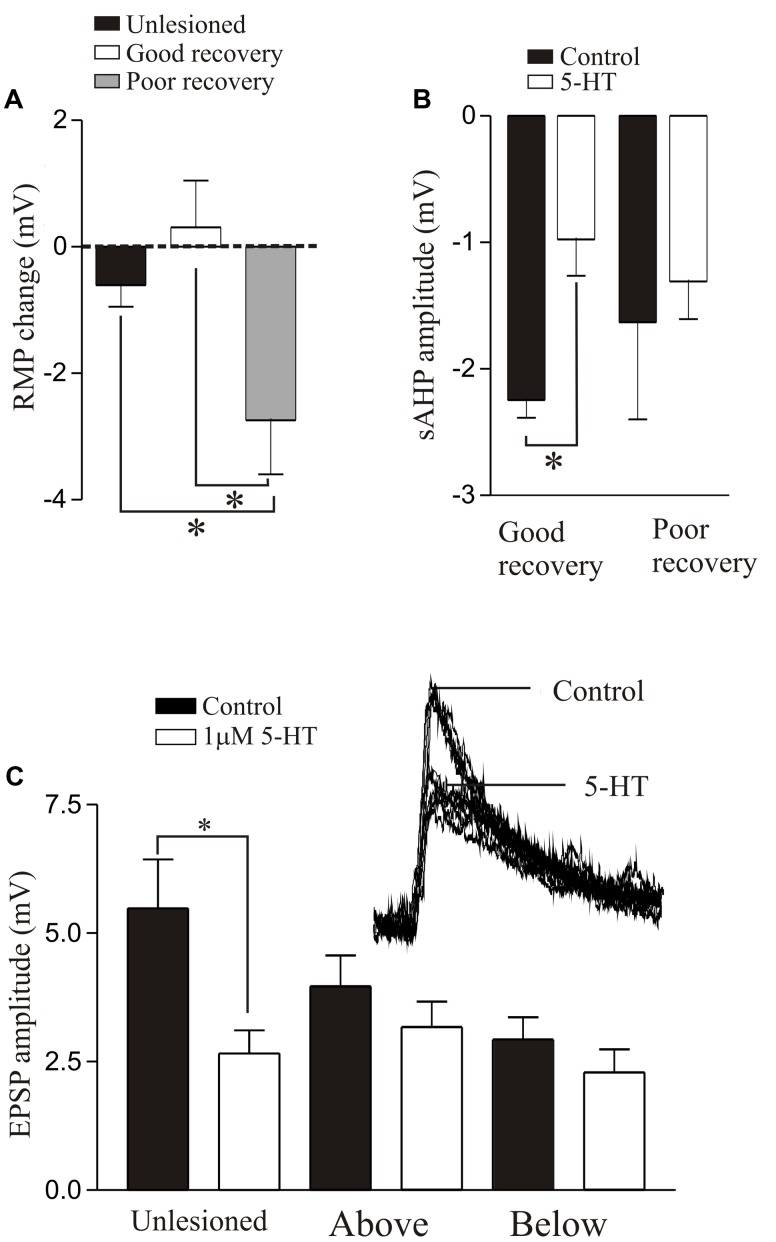
5-HT-mediated modulation after lesioning. **(A)** Graph showing the effects of 5-HT on the resting membrane potential in unlesioned animals, and lesioned animals that showed good and poor recovery. **(B)** Graph showing the effects of 5-HT on the sAHP in good and poor recovery: note the amplitude was only significantly reduced in animals that showed good recovery. **(C)** Graph showing the effects of 5-HT on glutamatergic synaptic inputs in unlesioned animals and lesioned animals above and below the lesion site. The inset shows the effect of 5-HT in an unlesioned spinal cord. Data from Becker and Parker (2015). No permission is required to reproduce this material. ^∗^Indicates statistical significance at *p* < 0.05.

The effects of 5-HT differed in unlesioned and lesioned animals that recovered well or poorly. 5-HT consistently evoked a hyperpolarization of the membrane potential in unlesioned animals (∼1 mV; see also Buchanan and Grillner, 1991). After lesioning 5-HT effects were more variable: with good recovery it typically depolarized the resting potential above and below the lesion site (Becker and Parker, 2015), but in animals that failed to recover it evoked a relatively large hyperpolarization below the lesion site (>2 mV, **Figure 8A**). This may negatively affect recovery by reducing the excitation:inhibition ratio from an optimal level (see above). 5-HT reduced the post-action potential sAHP and increased excitability in control animals and animals that recovered well (above and below the lesion site), but had no effect in animals that didn’t recover (**Figure 8B**), again suggesting a potential role in good recovery. A striking effect was that the 5-HT-mediated reduction of glutamatergic inputs, a highly consistent modulatory effect in unlesioned animals (Buchanan and Grillner, 1991; Parker and Grillner, 1999; Becker and Parker, 2015; McClelland and Parker, 2017), was absent above and below lesion sites (**Figure 8C**), suggesting that the synaptic effects of 5-HT are reduced after SCI.

**FIGURE 9:**
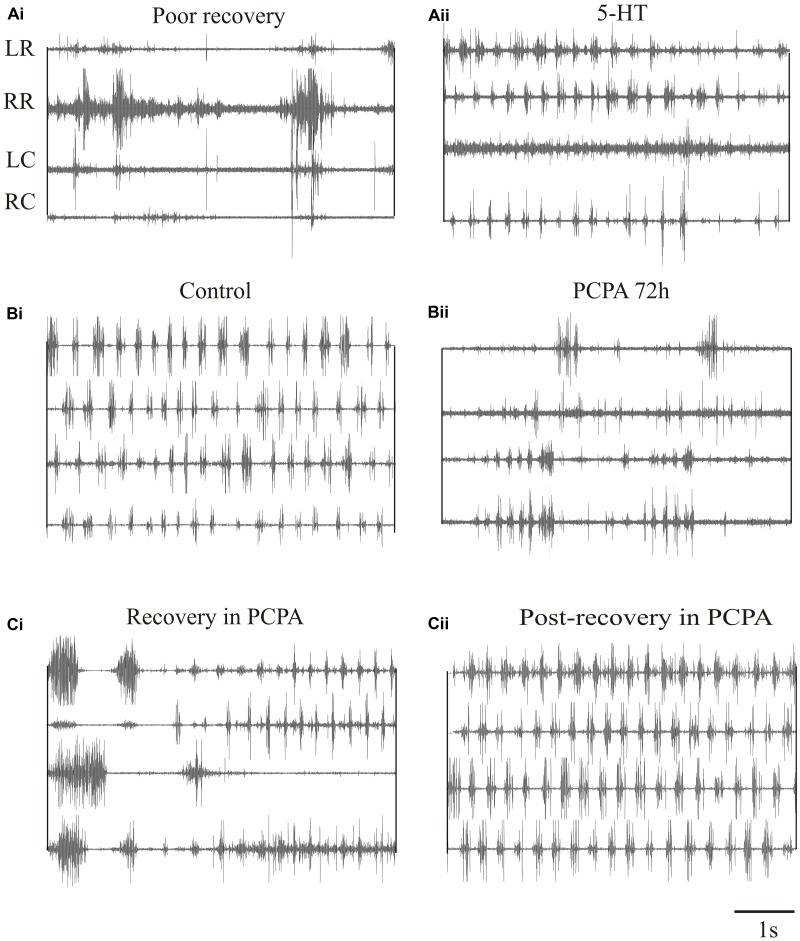
Effects of 5-HT on swimming. **(Ai,Aii)** EMG traces from swimming animals showing activity above and below the lesion site in an animal that failed to recover locomotor function, and the effects of 5-HT in improving activity in the same animal. **(Bi,Bii)** EMG traces showing activity in an unlesioned animal, and in the same animal after incubation in PCPA for 72 h to deplete 5-HT. **(Ci)** EMG traces showing the poor activity in an animal that was incubated in PCPA after lesioning. **(Cii)** EMG traces showing activity in a lesioned animal after incubation in PCPA after it had recovered. Note that after recovery incubation in PCPA was without effect. Data from Becker and Parker (2015). No permission is required to reproduce this material.

We have also examined the effects of 5-HT on locomotor behavior (Becker and Parker, 2015). 5-HT typically slows swimming in unlesioned animals and could improve its regularity. It had little effect in lesioned animals that recovered well (Becker and Parker, 2015), but could modestly improve swimming in animals that recover poorly (behavioral score change from 1/2 to stage 3; **Figures 9Ai,Aii**). However, 5-HT seems to be essential during recovery. Depleting 5-HT with p-chloro-phenylalanine (PCPA), a 5-HT synthesis inhibitor, markedly disrupted activity in unlesioned animals (**Figures 9Bi,Bii**), suggesting a necessary role for endogenous 5-HT in normal locomotor activity. Animals usually recovered normal swimming after removal of PCPA for 5 days (Becker and Parker, 2015). When lesioned animals were incubated in PCPA during recovery they consistently failed to recover locomotor function, suggesting a necessary role for 5-HT in recovery (**Figure 9Ci**). However, PCPA was without affect when it was applied after locomotor function had recovered (**Figure 9Cii**), suggesting that endogenous 5-HT becomes less important once recovery had occurred. These results are based on small numbers of animals. Given the variability of effects, especially after lesioning, further experiments are needed.

The simple conclusion from these analyses is that modulatory effects differ in lesioned animals. This seems to be a conserved effect (see Giroux et al., 2003 for differences after lesioning in cats), and suggests that drug effects after SCI cannot be predicted from effects in unlesioned animals. Even if this was unique to lamprey, assuming that a drug that targets a single transmitter system can restore function seems unlikely given the multiple transmitter systems and their potential interactions in the spinal cord (see above). An effective pharmacological approach after SCI will require understanding the functional state of locomotor networks and their inputs in the lesioned and unlesioned state, and the changes in transmitter systems and their effects.

## Conclusion

### What Have We Learnt from Lamprey?

(1) Regeneration is usually necessary for recovery, but as recovery can occur in its absence it may not be an absolute necessity. This does not negate the importance of promoting regeneration, but means that other approaches, for example electrical or pharmacological stimulation, could substitute for regeneration given optimal conditions.
(2) Regeneration is not restoration: regeneration is never complete, regenerated axons project ectopically, and regenerated synapses have a different ultrastructure. Even though pre-lesion functional properties are restored at regenerated synapses, this reflects changes in synaptic function.
(3) As inputs from individual regenerated axons match those in unlesioned animals, there is no compensation at individual synapses for the reduced descending drive caused by incomplete regeneration (cf. Frank, 2014). There may be little point making this compensation, as stronger activation of a limited pool of neurons will not necessarily translate into optimal activation of the whole sub-lesion network, will be energetically expensive (Howarth et al., 2012), and could cause excitotoxic damage.
(4) Compensation seems to occur in locomotor networks through wide-ranging changes in cellular and synaptic properties and synaptic reorganization. The net effect of the cellular and synaptic changes seems to be an increase in spinal cord excitability, although this requires balancing by a parallel increase in inhibition.
(5) Even though regeneration is usually necessary for recovery, it is not sufficient. Regeneration does not equal recovery: recovery instead depends on how altered regenerated inputs interact with altered sub-lesion networks.
(6) Proprioceptive inputs are potentiated after SCI. The lack of correlation between the sensory changes and recovery could suggest that sensory inputs have no influence on recovery, which seems unlikely given the powerful sensory entrainment/reflex effects (McClellan and Sigvardt, 1988), or that the effect of sensory inputs depends on their interaction with other components of the motor system. The latter seems likely given the association between sensory systems and locomotor networks (Pearson, 2000).

**FIGURE 10:**
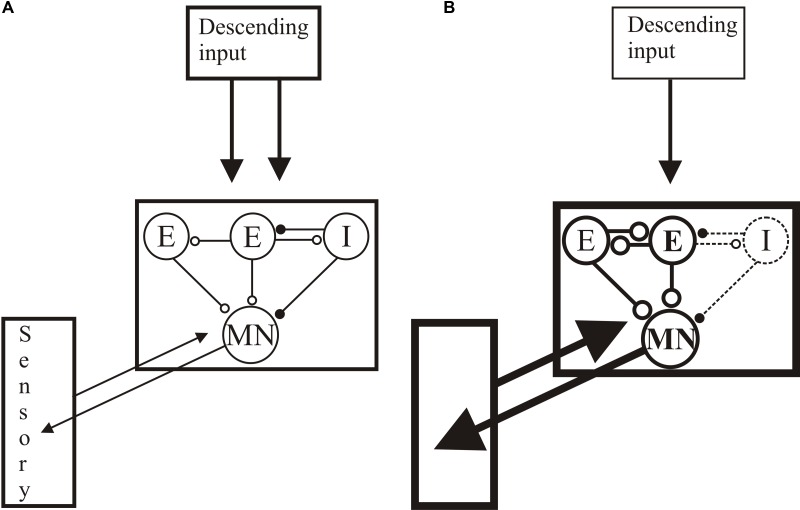
Summary of changes after lesioning. **(A)** The unlesioned network. Descending inputs project to the locomotor network. The network scheme is simplified to focus on known aspects, essentially limited to one half-center or hemisegmental network (open circles are glutamatergic synapses, filled circles are glycinergic synapses). Two hemisegmental networks in each spinal segment control activity on the left and right sides of the body, and are coupled by reciprocal inhibitory connections, the nature of which remains uncertain (Parker, 2006). The hemisegmental network contains EINs that provide glutamatergic inputs to other EINs, motor neurons, and the small ipsilateral inhibitory interneurons (SiIN): the latter provide feedback inhibition to the EINs and feedforward inhibition to motor neurons (this circuitry has been characterized in adults (Jia and Parker, 2016)). Movement is detected by proprioceptive edge cells that provide feedback to the locomotor network. **(B)** General summary of the changes after injury: see text for details of above/below, good/poor recovery, and larval/adult changes. Thicker lines represent increased activity, thinner reduced. Descending inputs to the spinal cord are reduced in number but individual connections are unaltered after lesioning. In the locomotor network there are changes in the cellular properties of EINs and motor neuron (larvae) and the connectivity and synaptic properties of the EINs (larvae and juveniles). Connections to the SiINs seem a key difference, with increased activity associated with poor recovery in larvae but with good recovery in adults. Sensory inputs are also increased after lesioning.

The restoration of normal locomotion after SCI thus reflects regeneration and functional changes in locomotor networks and sensory inputs (see **Figure 10**): the same behavioral output is generated by an anatomically and functionally different spinal cord. Recovery is thus more than reconnecting the two sides of the spinal cord. Sub-lesion changes may adapt the spinal cord to the reduced descending excitatory drive, while supra-lesion changes may generate stronger proprioceptive or mechanical responses to relay activity across the lesion site. Potentiated sensory feedback may in turn be needed to regulate the activity in these altered networks. While these are plausible responses to injury, we lack insight into their direct roles. Understanding their influence and underlying mechanisms will allow them to be targeted to improve functional recovery. Even if not targeted directly, as they will influence any regenerative, prosthetic, training or pharmacological input, understanding these changes is necessary to any rational intervention after SCI.

However, understanding these mechanisms and relating them to behavior is not trivial, even for simpler systems likes the lamprey (see Parker, 2010). It seems reasonable that the role of a component can be inferred by manipulating it and monitoring the effect on the system. However, this assumes that separate parts can be altered in isolation, an unlikely assumption in any feedforward and feedback (i.e., circular) system where even highly focused perturbations can cause effectively instantaneous changes at non-perturbed sites (Otchy et al., 2015). Even without these effects, loss of function when a component is removed only shows necessity, not sufficiency, while absence of an effect may reflect degeneracy or redundancy by compensatory adjustments rather than it being unnecessary.

### What Do We Need to Do: How Can Lower Vertebrates Help?

(1) *We need to understand the properties of regenerated synapses.* While synaptic properties will ultimately determine what regenerated axons will do, this aspect has received very little attention compared to axonal regeneration across lesion sites. The changes in the sub-lesion spinal cord mean that restoring a pre-lesion descending input will be unnecessary, unsuccessful, or even undesirable. We instead need to ensure that regenerated inputs make appropriate connections with sub and supra-lesion networks. Anatomical and functional connectivity arises in an orchestrated developmental process that may be difficult to recapitulate in adults (see Cardozo et al., 2017), but understanding how regenerated connections interact effectively with locomotor networks will provide options for interventions. *The analysis of regenerated connections is feasible in lamprey* (**Figure 2**), *and can identify features associated with successful synaptic re-integration that can be applied to mammalian analyses.*
(2) A core requirement is the need to determine whether the changes in spinal cord networks are beneficial or deleterious to recovery. Sub-lesion changes in locomotor networks and sensory systems are routine across systems. However, their roles are generally complex and unclear: even deleterious effects (e.g., spasticity) can be beneficial under some conditions (Adams and Hicks, 2005). The routine pharmacological block of these changes clinically after SCI may be inadvisable as it could remove potentially beneficial intrinsic effects: it is better to try to understand the changes and find ways to utilize them.
  The impact of any effect will depend on its intrinsic properties and how it interacts with the locomotor network. Identifying these changes and their impact on recovery is crucial, but can be difficult in mammals (Rybak et al., 2015). Lower vertebrates offer advantages. The lamprey allows cellular and synaptic effects to be examined. The zebrafish may be especially useful, as it offers the opportunity to combine molecular and physiological analyses (Ampatzis et al., 2014; Barreiro-Iglesias et al., 2015) that can identify functional changes and their influence on recovery.
(3) *We need to understand the interventions needed after SCI.* Self-organization through compensatory changes may underlie spontaneous recovery in lower vertebrates or neonatal mammals, but the limited recovery of adult mammals suggests that these mechanisms are insufficient, absent or, like the regenerative capacity of the spinal cord, actively suppressed. We need to understand how to intervene to trigger effects conducive to recovery, and when to intervene given that effects can be beneficial or deleterious depending on timing (Reiss et al., 2012). This could reflect state-dependent influences, possibly caused by secondary changes and compensations after injury. Sensory modification with locomotor training currently seems the best approach to SCI (D’Amico et al., 2014), and presumably drives the reorganization of spinal and other circuits (e.g., as in constraint-induced therapy after stroke; Kwakkel et al., 2015). However, training studies often lack standardized methods and assessment and have insufficient sample sizes. Improvements are also variable and modest (see Morawietz and Moffat, 2013). As with regeneration (Tuszynski and Steward, 2012) this may reflect the focus on a single aspect in an integrated system: interactive effects seem to offer greater promise (see Fong et al., 2009; Roy et al., 2012). The ultimate functional effects of any intervention will depend not just on the properties of the targeted component but on the state of the system it interacts with. *Rational interventions require understanding the role of changes in the spinal cord outlined above, and knowing how these can be manipulated to move the system into an optimal functional state.*
(4) *We need to understand the relevance of variability after SCI.* Parameters vary in spinal cord circuits (e.g., Parker, 2003a), and this seems to increase after lesioning (Cooke and Parker, 2009; Hoffman and Parker, 2011; Becker and Parker, 2015), the appearance of values three to four times the standard deviation suggestive of a power law relationship (Buzsaki and Mizuseki, 2014). Analyses sometimes reduce variability to improve statistical significance (see Steward et al., 2012; Tuszynski and Steward, 2012). While extraneous variables should be minimized, controlling system variables to maximize statistical effects is unlikely to identify strategies that translate to highly variable clinical situations. Disease states can show regular periodic activity indicative of a loss of complexity and variability (Mackey and Glass, 1977; West, 2010), suggesting that rather than being noise, variability is a signal that allows the system to explore a range of values in parameter space to optimize recovery (Ashby, 1958). This makes a “fix the numbers” approach that attempts to restore parameters to some average value unlikely to be successful (see Goldberger et al., 2000; Mutch et al., 2000; Volkmann et al., 2006). While not directly related to variability of functional parameters, introducing variability into locomotor training regimes resulted in a greater improvement in function (Shah et al., 2012). *Analyses of variability need the characterization of identified parameters pre and post-lesion. This is possible in mammalian systems* (Butt and Kiehn, 2003; Hinckley and Ziskind-Conhaim, 2006), *but is easier in lower vertebrate systems (e.g., of excitatory network interneurons in lamprey and zebrafish*; Parker, 2003a; Ampatzis et al., 2014). *Testing the role of variability requires that it is manipulated without changing mean values. This can be difficult to do experimentally in any system, but the characterization of variability after SCI will allow computational analyses.*

## Author Contributions

The author confirms being the sole contributor of this work and approved it for publication.

## Conflict of Interest Statement

The author declares that the research was conducted in the absence of any commercial or financial relationships that could be construed as a potential conflict of interest.
